# Optimal subsidy design for sports tourism under demand uncertainty: A multi-agent strategic interaction framework

**DOI:** 10.1371/journal.pone.0329682

**Published:** 2025-08-29

**Authors:** Yuanyuan Yin

**Affiliations:** University of South China, Hengyang, Hunan, China; Universidad Tecnica Federico Santa Maria, CHILE

## Abstract

This study develops a dynamic game model to analyze subsidy mechanisms for sports tourism integrated destination (STID), examining interactions between a core operator (major projects) and multiple supporting operators (ancillary services). It compares three scenarios: no subsidy, investment-based subsidies (core operator only), and visitor-driven subsidies (tied to tourist numbers). Results show investment-based subsidies increase core operator spending but not supporting operators’ investments, while boosting tourist numbers and supporting operators’ profits. Visitor-driven subsidies benefit all operators, increasing investments, profits and tourist volumes. When subsidy budgets are equal, visitor-driven subsidies yield better outcomes across all metrics if demand fluctuations remain stable. Demand shocks amplify both subsidy types’ effectiveness. Findings suggest visitor-driven subsidies better coordinate tourism ecosystems, with demand volatility enhancing subsidy impacts, while core and supporting operators require differentiated policy approaches due to asymmetric responses.

## 1 Introduction

The development of sports tourism integrated destination (STID) worldwide has fully demonstrated its key role in promoting regional economic growth, cultural prosperity, and achieving sustainable development goals. International renowned STIDs such as Queenstown (New Zealand), Bokala (Nepal), and Shamoni (France) are potent examples of this role. Queenstown, known for its adventure sports, generates an annual economic output of over 2.5 billion New Zealand dollars through its related projects, which strongly drives private investment in supporting services. Similarly, Pokhara, which features paragliding, saw a 35% increase in tourism revenue within five years.

In China, the promotion of national policies, such as the “Healthy China 2030” plan and the “Integrated Development of Sports and Tourism” initiative, is driving a thriving trend in the construction of STIDs. Several excellent STIDs have emerged successively, including Changbai Mountain Wanda Sports Tourism Scenic Area, Jiaozuo Taiji Sports Center Scenic Area, Kunming Ten Peaks Mountaineering Sports Tourism Route, and Rongjiang Seventy Two Villages Ethnic Sports Bullfighting Town.

The construction of STIDs not only enables consumers to enjoy physical and mental well-being through exercise but also has the potential to impact public health positively (Guironnet, 2023) [1]. It may indirectly reduce medical expenses, thereby contributing to economic development and enhancing comprehensive national strength, highlighting its significant external positive effects. From the perspective of economic principles, for projects with positive external effects, the government should provide subsidy support.

Especially for impoverished areas, if they can leverage their unique ethnic culture, topography, and other resource advantages to develop sports tourism, it will effectively promote rural revitalization and narrow the income gap between urban and rural areas. Therefore, the government must provide strong support for the development of sports tourism destinations through various means, such as subsidies [2].

There are various forms and effects in the specific implementation of subsidy policies. Subsidies based on investment, such as direct investment in infrastructure construction, can effectively stimulate the initial development of projects but often cannot guarantee long-term operational quality. This has been reflected in the practice of some mountain resorts in China [3]. Performance-based subsidies, such as reward mechanisms linked to tourist numbers or satisfaction, are more effective in maintaining service standards. Queenstown’s innovative “Tourist Night” subsidy indicator is a successful example. From this, optimizing the subsidy policy mechanism is crucial for enhancing the effectiveness of subsidies and improving the efficiency of fund utilization. In-depth research on the mechanisms of government subsidies for the construction of sports tourism destinations has significant practical implications.

Additionally, the uncertainty of demand poses significant challenges to the sustainable development of sports tourism destinations. Shamoni’s practice has shown that destinations with diverse activity combinations and flexible subsidy policies can better cope with demand fluctuations and maintain 85% of pre-crisis income levels during economic downturns (https://www.auvergnerhonealpes-tourisme.com).

Based on the above analysis and in-depth investigation of the construction practices of STIDs, this article constructs a differential game model involving multiple participants in the investment and operation of STIDs. This model fully considers the uncertainty of consumer demand and analyzes the optimal strategies and results of game participants in three different scenarios: no government subsidies, subsidy investment quantity, and subsidy investment performance (measured by the number of destination consumers). The aim is to provide a scientific basis for the government to formulate optimal subsidy strategies by studying micro-subject behavior.

Unlike previous studies that mainly focused on macro-level research, this study may have the following marginal contributions:

(1) Construct a dynamic game theory framework to deeply analyze the hierarchical relationship between core operators and supporting operators in the construction of STIDs, as well as the strategic interaction between the two under different subsidy systems.
(2) The first systematic comparison of the impact of investment-based and performance-based STID subsidy mechanisms on investment decisions of various stakeholders and overall target performance.
(3) Incorporate demand uncertainty into the analysis scope, quantify its impact, and determine the optimal adjustment path for the subsidy mechanism to enhance the system’s risk resistance capability.

The subsequent content of this article is as follows: Section 2 (2 Literature Review) provides a systematic review of relevant literature; Section 3 (3 Basic Assumptions) elaborates on the differential game model constructed in this article; Sections 4–5 (4 Model and Equilibrium Results and 5 Discussion on the Results) delve into the equilibrium results under different subsidy scenarios; Section 6 (6 Conclusions) explores the impact of policies and draws research conclusions.

## 2 Literature review

Existing scholarship provides foundational insights into three interrelated domains central to this study: the development of STIDs, investment behavior amid market volatility, and the efficacy of public subsidy mechanisms. By synthesizing these strands, we identify critical gaps this research seeks to address.

### 2.1 The development of STIDs

Recent scholarship has redefined STIDs as integrated ecosystems where athletic pursuits serve as primary attractions, seamlessly interconnected with hospitality offerings and cultural experiences.[4,5] This systemic perspective highlights the multidimensional nature of STID development, emphasizing the interdependence of various stakeholders and environmental factors.

A growing body of research underscores the critical role of community engagement in successful STID projects. Empirical studies by Chang et al. [6] and Ma et al. [7] demonstrate that local residents’ active participation and support significantly contribute to the long-term viability of STIDs, particularly in preserving cultural authenticity and ensuring social sustainability. This aligns with earlier findings on the importance of cross-regional collaboration, where Caffyn and Lutz [8] identified resource sharing and joint marketing as key drivers of STID competitiveness.

The ecological dimension of STID development has gained increasing attention in recent years. He and Wu [9] proposed an eco-centric development model, arguing that environmental conservation should form the foundation of sustainable STID planning. This perspective finds strong support in Zhao and Han’s [10] case study of the Yellow River Basin, which revealed significant synergies between sports tourism and ecological preservation.

From a consumer perspective, Tang et al. [11] developed a comprehensive evaluation framework measuring the coordination between sports, culture, and tourism elements, providing valuable tools for assessing STID integration quality. Complementing this, Wang and Chen. [12] employed regression analysis to identify optimal locations for rural STID development, offering data-driven insights for site selection decisions.

These studies collectively advance our understanding of STID development by: Emphasizing the ecosystem nature of modern STIDs; Validating the importance of stakeholder collaboration; Introducing ecological considerations into development models; Providing quantitative assessment tools for planning and evaluation.

However, gaps remain in understanding how these multidimensional factors interact dynamically, particularly in the context of rapidly changing market conditions and policy environments – an area this study seeks to address.

### 2.2 Investment decision-making under uncertainty

Theoretical advances in accurate options analysis and stochastic modeling have shed light on how uncertainty influences capital allocation patterns across industries. In the energy sector, Husain et al. [13] establish that policy ambiguity induces temporal clustering of green technology investments, while Delaney’s [14] stochastic dynamic programming model reveals The impact of Knight uncertainty on irreversible investments. In addition, Sun et al. [15] document how Chinese firms use technological hedging to mitigate policy risk.

The existing literature has certain limitations in its research scope and does not address investment issues in STIDs projects. In recent years, scholars such as Deng [16], Lv et al. [17], and Zhang and Yi [3] have taken a different approach by using differential game models to conduct in-depth analysis of investment and management issues in STID projects. Their research results provide a solid theoretical foundation for subsequent studies in this paper. However, there are still many issues that urgently need to be explored in the complex field of STID project investment and operation: Co-opetition dynamics between primary and secondary service providers; The moderating effect of demand-side volatility on joint investment returns.

### 2.3 Subsidy Policy Design

Subsidy mechanisms have evolved from blunt fiscal instruments to targeted performance levers. The automotive industry offers instructive cases: Jiang and Xu’s [18] difference-in-differences analysis of Chinese EV subsidies reveals that output-linked incentives outperform pure capital injections in stimulating R&D productivity. Similarly, Asgarian et al. [19] demonstrate how Sweden’s carbon tax-subsidy hybrid achieved greater emissions reduction than either policy in isolation. Previous research on subsidy policies has not addressed the issue of subsidies for STIDs. Recently, Lv et al.[17] and Zhang and Yi [3] have respectively focused on the research of investment amount subsidies and investment performance subsidies for STID, and deeply analyzed the roles of these two subsidy methods in the development process of STID projects. Research has found that both investment amount subsidies and investment performance subsidies can promote the development of STID projects to a certain extent. However, a key question remains unanswered: which of these two subsidy methods is more advantageous? This question is like a “black box” that urgently needs to be further unraveled. This study advances the discourse by introducing a game-theoretic framework that addresses these gaps through its examination of multi-agent strategic interactions in STIDs under demand uncertainty.

## 3 Basic assumptions

Consider a local government’s decision to develop an STID project. According to China’s practice, the local government generally entrusts a large investor to be responsible for the investment and operation of major projects, such as infrastructure and sports venues. At the same time, through investment promotion or spontaneous investment by investors, some small investors or individual businesses also participate in the main project, engaging in activities such as catering, accommodation, and small commodity sales. We refer to these two types of investment operators as “core operator” and “supporting operator”, respectively. Among them, there is only one core operator. Moreover, there are n supporting operators. For convenience, it is further assumed that they are homogeneous.

According to the principle that investment increases capital and depreciation decreases capital, as well as related studies such as Jørgensen and Zaccour [20] and Zhang and Yi [3], the capital operated by two types of investors exhibits the following dynamics:


$$\frac{dk_{1}(t)}{dt}=u_{1}(t)-\delta_{1}k_{1}(t);\frac{dk_{2i}(t)}{dt}=u_{2i}(t)-\delta_{2}k_{2i}(t)$$ (1)

where, $k_{1}(t)$ and $k_{2i}(t)$ respectively describe the capital levels of the core investor and supporting investors i(i=1,2,⋯,n) at time t. And their initial levels are $k_{1}(0)=0$, $k_{2i}(0)=0$. $u_{1}(t)$ and $u_{2i}(t)$ are the investment levels of the two types of investors at time t. $\delta_{j}>0$ ( j=1,2) indicate the depreciation rates of two types of capital.

The investment cost typically follows the law of marginal increase, meaning that as the investment scale expands, the unit cost increases accordingly. Because investors tend to prioritize low-cost investments, as they continue to invest, the availability of low-cost opportunities decreases, leading to increased investment difficulty and costs. According to Deng et al. [16] and Lv et al. [17], the costs of two types of investors can be represented by the following quadratic function: i.e., $u_{1}^{2}/2,\;u_{2i}^{2}/2$.

Next, we survey the number of tourists and consumption demand for this STID. According to Zhang and Yi [3]‘s research, factors that affect demand can be classified into two categories: price factors and nonprice factors.

In terms of price factors, consumers will consider multiple aspects of price comprehensively. On the one hand, the price of the core project is crucial, and the charging situation of sports such as rock climbing, skiing, and skydiving is often the first concern for participants. On the other hand, the prices of supporting projects, such as catering and accommodation, cannot be ignored, as tourists cannot simply participate in sports without addressing their food and accommodation needs. Therefore, the prices of various projects will affect consumer demand.

From the perspective of nonprice factors, project size may be the most important influencing factor. The larger the scale, the more diverse the experiential projects that consumers can choose from and the stronger the attraction to tourists. Moreover, the scale will also impact the destination’s popularity. Therefore, whether it is a core project or a supporting project, its scale will have an impact on consumer demand.

In addition, some uncertain factors should not be underestimated. For example, factors such as economic prosperity, weather conditions, government policy adjustments, and epidemics are sudden and unpredictable, which may, to some extent, suppress tourists’ enthusiasm for STID consumption.

The above analysis leads to the following demand functions:


$$q(t)=[a-p_{1}(t)-p_{2i}(t)+\varepsilon ](\alpha_{1}k_{1}(t)+\alpha_{2}nk_{2i}(t))$$ (2)

The above function model reveals that price factors and non-price factors jointly affect demand through multiplication, which aligns with the relevant research results of Yi et al. [21] and Jiang and Xu [18].

From the perspective of price factors, both the price $p_{1}(t)$ of core projects and the price $p_{2i}(t)$ of supporting projects will hurt demand, which is consistent with the classical demand principle. In this model, the potential demand is set as a.

In terms of nonprice factors, the capital size of the two types of projects is the key influencing factor. The capital scale $k_{1}(t)$ of the core project and the capital scale $k_{2i}(t)$ of the supporting project both have a favorable driving effect on demand [3]. We use parameters $\alpha_{1}$ and $\alpha_{2}$ to quantify the impact of these parameters on demand, thereby describing the relationship between scale factors and demand more accurately.

In addition, the model also introduces a random perturbation term, ε, to characterize the uncertainty of demand. Its density function is denoted as f(ε), and the mean of the random perturbation term is 0, following a uniform distribution within the interval $\left[\varepsilon_{\min},\varepsilon_{\max}\right]$.

Finally, let us explore the government’s subsidy policies for STID projects. From multiple perspectives, including promoting national health, alleviating positive externalities, and fostering economic development, particularly in poverty-stricken areas, the government (generally referring to central and provincial governments) typically provides subsidies and support for STID projects. According to survey data, such subsidy amounts may account for about 30% of the total investment in core projects.

This article presents two subsidy models to explore in depth which subsidy method can achieve the best results. One is to provide subsidies based on the investment amount of core projects, which is quite common in reality. Secondly, it is based on investment performance, that is, implementing subsidies according to the size of consumers. In addition, for a more precise analysis, this article sets the situation where the government does not provide subsidies as a benchmark reference. Based on this, this article constructs three models around three scenarios: “no subsidy,” “subsidy based on investment quantity,” and “subsidy based on investment performance consumer scale.” In addition, to compare the effects of the two subsidy methods, our government’s expenditure on the two subsidies is equal, that is, $T=\int_{0}^{t}\tau_{1}u_{1}^{2}(t)/2dt=\int_{0}^{t}\tau_{2}q(t)dt$. Among them, $\tau_{1}$ and $\tau_{2}$ are the subsidy rates under two subsidy modes.

## 4 Model and Equilibrium Results

In this section, we use N, PI, and NS to represent three scenarios: no subsidy, subsidy investment quantity, and subsidy investment performance.

The models for three scenarios are shown in Table 1:

**Table 1:** The models for three scenarios.

Model	the profit functions
N	$\underset{p_{1},u_{1}}{\max}\pi_{1}(t)=\int_{0}^{\infty }e^{-\rho t}[p_{1}(t)q(t)-u_{1}^{2}(t)/2]dt;\;\underset{p_{2i},u_{2i}}{\max}\pi_{2i}(t)=\int_{0}^{\infty }e^{-\rho t}[p_{2i}(t)q(t)-u_{2i}^{2}(t)/2]dt$
PI	$\underset{p_{1},u_{1}}{\max}\pi_{1}(t)=\int_{0}^{\infty }e^{-\rho t}[p_{1}(t)q(t)-(1-\tau_{1})u_{1}^{2}(t)/2]dt;\;\underset{p_{2i},u_{2i}}{\max}\pi_{2i}(t)=\int_{0}^{\infty }e^{-\rho t}[p_{2i}(t)q(t)/n-u_{2i}^{2}(t)/2]dt$;
NS	$\underset{p_{1},u_{1}}{\max}\pi_{1}(t)=\int_{0}^{\infty }e^{-\rho t}[p_{1}(t)q(t)-u_{1}^{2}(t)/2+q(t)\tau_{2}]dt;\;\underset{p_{2},u_{2}}{\max}\pi_{1}(t)=\int_{0}^{\infty }e^{-\rho t}[p_{2}(t)q(t)-u_{2}^{2}(t)/2]dt$;

Among them, ρ is the risk-free rate, which is the discount rate.The constraints of the above three models are:


{\raise0.7ex{d{k_1(t)\)}/dk1(t)dt\nulldelimiterspace\lower0.7ex\({dt\)}}=u1(t)−δ1k1(t,k1(0)=0\raise0.7ex{d{k_1(t)\)}/dk1(t)dt\nulldelimiterspace\lower0.7ex\({dt\)}}=u2i(t)−δ2k2i(t,k2(0)=0

The optimal strategies are shown in Table 2:

**Table 2:** The optimal decision.

Model	Optimal strategy
N	$p_{1}^{N*}=\frac{a+\varepsilon }{3}$; $p_{2i}^{N*}=\frac{a+\varepsilon }{3}$; $u_{1}^{N*}=\frac{{\left(a+\varepsilon \right)}^{2}\alpha_{1}}{9(1+\delta_{1})}$; $u_{2i}^{N*}=\frac{{\left(a+\varepsilon \right)}^{2}\alpha_{2}}{9(1+\delta_{2})}$
PI	$p_{1}^{PI*}=\frac{a+\varepsilon }{3}$; $p_{2i}^{PI*}=\frac{a+\varepsilon }{3}$; $u_{1}^{PI*}=\frac{{\left(a+\varepsilon \right)}^{2}\alpha_{1}}{9(1-\tau_{1})(1+\delta_{1})}$; $u_{2i}^{PI*}=\frac{{\left(a+\varepsilon \right)}^{2}\alpha_{2}}{9(1+\delta_{2})}$
NS	$p_{1}^{NS*}=\frac{a-2\tau_{2}+\varepsilon }{3}$; $p_{2i}^{NS*}=\frac{a+\tau_{2}+\varepsilon }{3}$; $u_{1}^{NS*}=\frac{{\left(a+\tau_{2}+\varepsilon \right)}^{2}\alpha_{1}}{9(1+\delta_{1})}$; $p_{2i}^{NS*}=\frac{{\left(a+\tau_{2}+\varepsilon \right)}^{2}\alpha_{2}}{9(1+\delta_{2})}$

The equilibrium results under the optimal decision are shown in Table 3:

**Table 3:** The equilibrium results.

Model	the stabilized values of product quality level and e-commerce platform goodwill
N	$q^{N*}=(a+\varepsilon )(\alpha_{1}k_{1}^{N*}(t)+\alpha_{2}nk_{2i}^{N*}(t))/3$; $k_{1}^{N*}(t)=k_{1}^{\infty N}-k_{1}^{\infty N}e^{-\delta_{1}t}$; $k_{1\infty }^{N*}=(a+\varepsilon {)}^{2}\alpha_{1}/\left[9(1+\delta_{1})\delta_{1}\right]$; $k_{2i}^{N*}(t)=k_{2i\infty }^{N}-k_{2i\infty }^{N}e^{-\delta_{2}t}$; $k_{2i\infty }^{N*}=(a+\varepsilon {)}^{2}\alpha_{2}/\left[9(1+\delta_{2})\delta_{2}\right]$; $\pi_{1}^{N*}=e^{-\rho t}(m_{1}^{N*}k_{1}^{N*}+m_{2}^{N*}k_{2}^{N*}+m_{3}^{N*})$; $\pi_{2i}^{N*}=e^{-\rho t}(r_{1}^{N*}k_{1}^{N*}+r_{2}^{N*}k_{2}^{N*}+r_{3}^{N*})$; $J_{total}^{N*}=\pi_{1}^{N*}+n\pi_{2i}^{N*}$
PI	$q^{PI*}=(a+\varepsilon )(\alpha_{1}k_{1}^{PI*}(t)+\alpha_{2}nk_{2i}^{PI*}(t))/3$; $k_{1}^{PI*}(t)=k_{1\infty }^{PI}-k_{1\infty }^{PI}e^{-\delta_{1}t}$; $k_{1\infty }^{PI*}=(a+\varepsilon {)}^{2}\alpha_{1}\left[9(1-\tau_{1})(1+\delta_{1})\delta \right]$; $k_{2i}^{PI*}(t)=k_{2i\infty }^{PI}-k_{2i\infty }^{PI}e^{-\delta_{2}t}$; $k_{2i\infty }^{PI*}=(a+\varepsilon {)}^{2}\alpha_{2}/\left[9(1+\delta_{2})\delta_{2}\right]$; $\tau_{1}^{*}=18(1+\delta_{1})T/\left[{\left(a+\varepsilon \right)}^{2}\alpha_{1}t+18(1+\delta_{1})T\right]$; $\pi_{1}^{PI*}=e^{-\rho t}(m_{1}^{PI*}k_{1}^{PI*}+m_{2}^{PI*}k_{2i}^{PI*}+m_{3}^{PI*})$; $\pi_{2i}^{PI*}=e^{-\rho t}(r_{1}^{PI*}k_{1}^{PI*}+r_{2}^{PI*}k_{2i}^{PI*}+r_{3}^{PI*})$; $J_{total}^{PI*}=\pi_{g}^{PI*}+n\pi_{2i}^{PI*}$
NS	$q^{NS*}=(a+\tau_{2}+\varepsilon )(\alpha_{1}k_{1}^{NS*}(t)+\alpha_{2}nk_{2i}^{NS*}(t))/3$; $\pi_{1}^{NS*}=e^{-\rho t}(m_{1}^{NS*}k_{1}^{NS*}+m_{2}^{NS*}k_{2i}^{NS*}+m_{3}^{NS*})$; $\pi_{2i}^{NS*}=e^{-\rho t}(r_{1}^{NS*}k_{1}^{NS*}+r_{2}^{NS*}k_{2i}^{NS*}+r_{3}^{NS*})$; $J_{total}^{NS*}=\pi_{1}^{NS*}+n\pi_{2i}^{NS*}$; $\tau_{2}^{*}=\frac{T}{\frac{a+\tau_{2}+\varepsilon }{3}(\frac{\alpha_{1}u_{1}^{NS}}{\delta_{1}}(t+\frac{e^{-\delta_{1}t}-1}{\delta_{1}})+\frac{{\left(a+\tau_{2}+\varepsilon \right)}^{2}n{\alpha_{2}}^{2}}{9\delta_{2}(1+\delta_{2})}(t+\frac{e^{-\delta_{2}t}-1}{\delta_{2}}))}$

where, $m_{1}^{N*}=m_{1}^{PI*}=\frac{{\left(a+\varepsilon \right)}^{2}\alpha_{1}}{9(1+\delta_{1})}$, $m_{2}^{N*}=m_{2}^{PI*}=\frac{{\left(a+\varepsilon \right)}^{2}\alpha_{2}n}{9(1+\delta_{2})}$, $m_{3}^{N*}=(m_{1}^{N*}{)}^{2}/2+m_{2}^{N*}r_{2}^{N*}$, $r_{1}^{N*}=r_{1}^{PI*}=\frac{{\left(a+\varepsilon \right)}^{2}\alpha_{1}}{9n(1+\delta_{1})}$, $r_{2}^{N*}=r_{2}^{PI*}=\frac{{\left(a+\varepsilon \right)}^{2}\alpha_{2}}{9(1+\delta_{2})}$, $r_{3}^{N*}=(r_{2}^{N*}{)}^{2}/2+m_{1}^{N*}r_{1}^{N*}$, $m_{3}^{PI*}=(\frac{1}{\left(1-\tau_{1}\right)}-\frac{1}{2{\left(1-\tau_{1}\right)}^{2}})(m_{1}^{PI*}{)}^{2}+m_{2}^{PI*}r_{2}^{PI*}$, $r_{3}^{PI*}=(r_{2}^{PI*}{)}^{2}/2+\frac{1}{\left(1-\tau_{1}\right)}m_{1}^{PI*}r_{1}^{PI*}$,

$m_{1}^{NS*}=\frac{{\left(a+\tau_{2}+\varepsilon \right)}^{2}\alpha_{1}}{9(1+\delta_{1})}$, $m_{2}^{NS*}=\frac{{\left(a+\tau_{2}+\varepsilon \right)}^{2}\alpha_{2}n}{9(1+\delta_{2})}$, $m_{3}^{NS*}=(m_{1}^{NS*}{)}^{2}/2+m_{2}^{NS*}r_{2}^{NS*}$;

$r_{1}^{NS*}=\frac{{\left(a+\tau_{2}+\varepsilon \right)}^{2}\alpha_{1}}{9n(1+\delta_{1})}$, $r_{2}^{NS*}=\frac{{\left(a+\tau_{2}+\varepsilon \right)}^{2}\alpha_{2}}{9(1+\delta_{2})}$, $r_{3}^{NS*}=(r_{2}^{NS*}{)}^{2}/2+m_{1}^{NS*}r_{1}^{NS*}$.

## 5 Discussion on the results

### 5.1 Sensitivity

Corollary 1**:** $\frac{\partial p_{1}^{j*}}{\partial \varepsilon }=\frac{\partial p_{2}^{j*}}{\partial \varepsilon }=\frac{1}{3}$, $\frac{\partial q^{j*}}{\partial \varepsilon }>0$, $\frac{\partial u_{1}^{j*}}{\partial \varepsilon }>0$, $\frac{\partial k_{1}^{j*}}{\partial \varepsilon }>0$, $\frac{\partial u_{2i}^{j*}}{\partial \varepsilon }>0$, $\frac{\partial k_{2i}^{j*}}{\partial \varepsilon }>0$, where, j=N,PI,NS.

This conclusion indicates that in the three analyzed scenarios, the random disturbance term of demand will have a positive impact on prices, consumer demand, investment behavior of both types of investors, and capital size. Specifically, when favorable demand disturbances occur, such as an increase in consumer income levels or the introduction of government policies to encourage national health, these positive factors act as catalysts, driving synchronous growth in prices, consumer demand, and investment scale. This positive cycle is beneficial for the flourishing development of STID projects. On the contrary, if encountering negative disturbances, such as the impact of the epidemic or economic downturn, the above-mentioned variables will be impacted and undergo unfavorable changes. Price fluctuations, shrinking demand, and reduced investment hinder the smooth progress and long-term development of STID projects.

**Corollary 2:** The increase in subsidy rates for the core operator investments has the following impact:

(1) $\frac{\partial p_{1}^{PI*}}{\partial \tau_{1}}=\frac{\partial p_{2}^{PI*}}{\partial \tau_{1}}=\frac{\partial u_{2i}^{PI*}}{\partial \tau_{1}}=\frac{\partial k_{2i}^{PI*}}{\partial \tau_{1}}=0$, $\frac{\partial u_{1}^{PI*}}{\partial \tau_{1}}>0$, $\frac{\partial k_{1}^{PI*}}{\partial \tau_{1}}>0$. $\frac{\partial q^{PI*}}{\partial \tau_{1}}>0$.
(2) If $0<\tau_{1}<\tau_{1c}$, $\frac{\partial \pi_{1}^{PI*}}{\partial \tau_{1}}>0$; if $\tau_{1c}<\tau_{1}<1$, $\frac{\partial \pi_{1}^{PI*}}{\partial \tau_{1}}<0$.
(3) $\frac{\partial \pi_{2i}^{PI*}}{\partial \tau_{1}}>0$. where $\tau_{1c}=\frac{\delta_{1}}{1+\delta_{1}}$.

It reveals that providing investment quantity subsidies for the core operator can effectively incentivize it to increase its investment and drive the expansion of the corresponding capital scale. However, it does effect the investment of the supporting operators. At the price level, both parties’ service pricing has remained stable without any fluctuations. At the same time, the demand in the consumer market is showing a growing trend, and the profit level of auxiliary operators is increasing accordingly. What is particularly noteworthy is that the profit changes of the core operator exhibits non-linear characteristics: when the subsidy rate is below a certain critical threshold, its profits increase with the increase of subsidies, But once the subsidy rate exceeds this threshold, profits will not increase but decrease, and this phenomenon deserves further research and exploration.

As the subsidy rate increases, the investment scale of the core operator also expands, resulting in it having to bear increasing marginal costs. On the other hand, with the increase in investment and corresponding capital, service prices and consumer demand will also rise in tandem, thereby promoting the synchronized growth of service income. Therefore, for the core operator, the increase in subsidy rates has resulted in its profits being affected by both opposing forces: revenue growth and cost increases. Specifically, when the subsidy rate is below a certain threshold, the positive effect of income growth prevails, resulting in increased profits. When the subsidy rate exceeds this threshold, the negative impact of cost increase outweighs the positive effect of income growth, leading to a decrease in profits.

Auxiliary operators will not increase their investment due to this subsidy policy. However, they can benefit from the “east wind” of rising service prices and increased consumer demand resulting from the core operator’s investment. Therefore, the profits of auxiliary operators will inevitably increase with the rise in subsidy rates.

**Corollary 3:** The impact of the increase in subsidy rates for consumer scale subsidies is as follows: $\frac{\partial p_{1}^{NS*}}{\partial \tau_{2}}=-\frac{2}{3}$, $\frac{\partial p_{2}^{NS*}}{\partial \tau_{2}}=\frac{1}{3}$, $\frac{\partial q^{NS*}}{\partial \tau_{2}}>0$, $\frac{\partial u_{1}^{NS*}}{\partial \tau_{2}}>0$, $\frac{\partial u_{2i}^{NS*}}{\partial \tau_{2}}>0$, $\frac{\partial k_{1}^{NS*}}{\partial \tau_{2}}>0$, $\frac{\partial k_{2i}^{NS*}}{\partial \tau_{2}}>0$, $\frac{\partial \pi_{1}^{NS*}}{\partial \tau_{2}}>0$, $\frac{\partial \pi_{2i}^{NS*}}{\partial \tau_{2}}>0$.

The above research results are enlightening. When implementing subsidy policies targeting the number of consumers, as the subsidy rate gradually increases, the service prices of the core operator and supporting operators show an opposite trend: the service prices of core operators decrease while the prices of supporting operators increase. At the same time, the consumer base has significantly expanded.

The logic behind this phenomenon is that when the core operator is subsidized based on consumer size, it can increase investment and expand its capital scale. The expansion of capital scale leads to the expansion of consumer scale. In addition, it will also cooperate with capital expansion strategies and adopt price reduction and promotion measures to stimulate consumer demand further, thereby earning more service revenue and government subsidies. Although price reduction may lead to a decrease in marginal income, overall, the positive effect of income increase is more significant and dominates.

For auxiliary operators, the price reduction and promotion strategy of the core operator has dramatically expanded the size of the consumer market. In this context, they can take the opportunity to increase service prices in order to gain more profits.

The research results also indicate that when the subsidy rate of performance subsidies increases, the investment and capital levels of both core and auxiliary operators show an upward trend.

The reason for the increase in investment by the core operator is that it has received more subsidy funds. The increase in investment by auxiliary operators is partly due to the rise in service prices, which provides them with more abundant funds for investment. On the other hand, to meet the higher service demands brought about by the expansion of the consumer base, they must also increase investment to enhance their service capabilities.

Finally, the increase in profits for both types of operators is due to the positive effect of the increased subsidy rates, which outweighs the negative effect of higher investment expenditures.

### 5.2 Comparison of Results Between Three Scenarios

#### 5.2.1 Compare N and PI.

**Corollary 4:** $p_{1}^{PI*}=p_{1}^{N*}$, $p_{2}^{PI*}=p_{2}^{N*}$, $q^{PI*}>q^{N*}$, $u_{1}^{PI*}>u_{1}^{N*}$, $u_{2i}^{PI*}=u_{2i}^{N*}$, $k_{1}^{PI*}>k_{1}^{N*}$, $k_{1\infty }^{PI}(t)>k_{1}^{\infty N}(t)$, $k_{2i}^{PI*}=k_{2i}^{N*}$, $k_{2i\infty }^{PI}(t)=k_{2i\infty }^{N}(t)$

The above results are similar to Corollary 1, therefore no additional description or explanation is needed.

**Corollary 5**: The following relationships exist:

(1)if $\tau_{1}<\tau_{1a}$, $V_{1}^{NPI}=\pi_{1}^{PI*}-\pi_{1}^{N*}>0$, $\frac{\partial V_{1}^{NPI}}{\partial \tau_{1}}>0$; (2) if $\tau_{1}>\tau_{1a}$, $V_{1}^{NPI}=\pi_{1}^{PI*}-\pi_{1}^{N*}<0$, $\frac{\partial V_{1}^{NPI}}{\partial \tau_{1}}<0$. (3) $V_{2i}^{NPI}=\pi_{2i}^{PI*}-\pi_{2i}^{N*}>0$, $\frac{\partial V_{1}^{NPI}}{\partial \tau_{1}}>0$. (4) If $\tau_{1}<\tau_{1b}$, $V_{total}^{NPI}=J_{total}^{PI*}-J_{total}^{N*}>0$, $\frac{\partial V_{total}^{NPI}}{\partial \tau_{1}}>0$; if $\tau_{1}>\tau_{1b}$, $V_{total}^{NPI}=J_{total}^{PI*}-J_{total}^{N*}<0$, $\frac{\partial V_{total}^{NPI}}{\partial \tau_{1}}<0$. (5) If $\tau_{1}<\tau_{1d}$, $\frac{\partial V_{total}^{NPI}}{\partial \tau_{1}}>0$; if $\tau_{1}>\tau_{1d}$, $\frac{\partial V_{total}^{NPI}}{\partial \tau_{1}}<0$. Where $\tau_{1a}=\frac{2(1-e^{-\delta_{1}t})}{2(1-e^{-\delta_{1}t})+\delta_{1}}$, $\tau_{1b}=\frac{4(1-e^{-\delta_{1}t})+2\delta_{1}}{4(1-e^{-\delta_{1}t})+3\delta_{1}}$, $\tau_{1c}=\frac{\delta_{1}}{1+\delta_{1}}$. $\tau_{1d}=\frac{2(1-e^{-\delta_{1}t})+\delta_{1}}{2(1-e^{-\delta_{1}t}+\delta_{1})}$.

Corollary 5 illustrates the difference in profits between parties with and without subsidies under the first type of subsidy. Based on this, there are four thresholds for subsidy rates, namely $\tau_{1a}$, $\tau_{1b}$, $\tau_{1c}$, and $\tau_{1d}$.

Among them, (1) shows that when subsidizing investments in the core operator, the situation differs from the conventional perception, and the operator’s net return is not necessarily increasing. Specifically, when the subsidy rate is below a certain threshold ( $\tau_{1a}$), the net return will increase with the increase of the subsidy rate. However, once the subsidy rate exceeds this threshold, the net return will decrease as the subsidy rate further increases.

Under such subsidy mechanisms, the core operator can obtain maximum benefits at a moderate subsidy rate level rather than excessively high subsidy rates. As for the reasons behind it, we have already elaborated on them in detail in the previous section and will not repeat them here.

The (2) in it shows that under the first type of subsidy, as the subsidy rate increases, the profit difference between affiliated operators with and without subsidies continues to rise with the subsidy rate, which is similar to inference 2.

The (3) in it examines the change in profit increment due to subsidies with the subsidy rate under the first type of subsidy from the overall perspective of both core and subsidiary operators. It shows that there is a threshold of $\tau_{1b}$, and when the subsidy rate is below this threshold, the overall profit increment is positive. Otherwise, it is negative. The reason is also due to the existence of threshold boundaries for the profit increment of the core operator, although there is no such threshold boundary for affiliated operators.

The difference between (4) and (3) in Corollary 5 is that the former examines the changes in profits under the first subsidy, while the latter analyzes the changes in profit increments. From this, we discovered two ranges of changes in overall profit caused by a new threshold $\tau_{1d}$. Among them, profits increase in the first interval, while profits decrease in the second interval.

**Fig 1:**
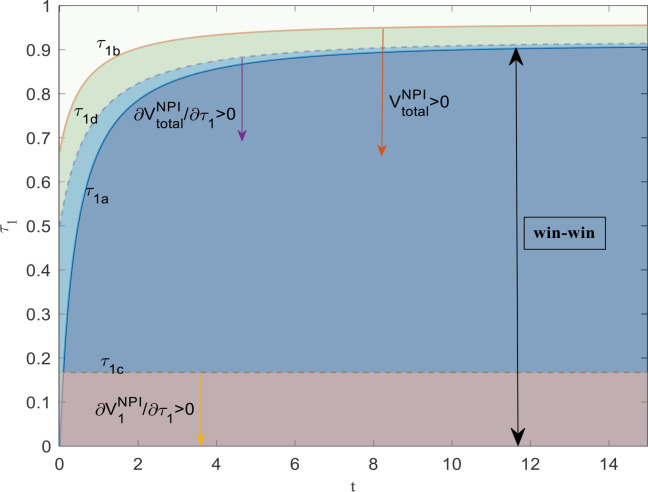
Profit improvement of the two participants and overall in investment subsidy rate.

Fig. 1 also shows that the maximum improvement in net returns for STID occurs when the subsidy rate is $\tau_{1d}$. Because below that line, $\partial V_{total}^{NPI}/\partial \tau_{1}>0$; above that line, $\partial V_{total}^{NPI}/\partial \tau_{1}<0$.

The insight from the above conclusion is that for the core operator and the overall STID, a higher subsidy rate for investment is not necessarily better. The subsidy rate for maximizing profit is at an appropriate height. When the subsidy rate exceeds the threshold, a higher subsidy rate has adverse effects (the thresholds for the two are different). This discovery provides a valuable reference for government departments to design subsidy policy mechanisms.

#### 5.2.2 Compare N and NS.

**Corollary 6**: $p_{1}^{NS*}<p_{1}^{N*}$, $p_{2}^{NS*}>p_{2}^{N*}$, $q^{NS*}>q^{N*}$, $u_{1}^{NS*}>u_{1}^{N*}$, $u_{2i}^{NS*}>u_{2i}^{N*}$, $k_{1}^{NS*}>k_{1}^{N*}$, $k_{1\infty }^{NS}(t)>k_{1}^{\infty N}(t)$, $k_{2i}^{NS*}>k_{2i}^{N*}$, $k_{2i\infty }^{NS}(t)>k_{2i\infty }^{N}(t)$.

The above results can be considered as another expression of Corollary 3, so we will not repeat the discussion here.

**Corollary 7:** $V_{1}^{NNS}=\pi_{1}^{NS*}-\pi_{1}^{N*}>0$, $V_{2i}^{NNS}=\pi_{2i}^{NS*}-\pi_{2i}^{N*}>0$, $V_{total}^{NNS}=J_{total}^{NS*}-J_{total}^{N*}>0$.

(1) $\frac{\partial V_{total}^{NNS}}{\partial \tau_{2}}=\frac{\partial V_{total}^{NNS}}{\partial \varepsilon }>0$, $\frac{\partial V_{2i}^{NNS}}{\partial \tau_{2}}>0$, $\frac{\partial V_{1}^{NNS}}{\partial \tau_{2}}>0$.

Corollary 7 and Corollary 5, respectively, describe the impact of two subsidy methods, namely subsidizing core operator investment and subsidizing STID consumption scale, on the improvement of STID operators and overall profits. It can be seen from this that the two subsidies have different effects on improving profits.

Corollary 5 demonstrates that a threshold exists for the subsidy rate applicable to core operators’ investments, as well as for the overall profit improvement of all parties. In the first range of the threshold, the profit improvement increases with the subsidy rate, whereas in the second range, it decreases with the subsidy rate. However, Corollary 7 shows that there is no interval divided by a threshold when subsidizing operational performance or consumer size, and the overall profit improvement increases monotonically with the subsidy rate.

#### 5.2.3 Compare PI and NS.

**Corollary 8:** $p_{1}^{NS*}<p_{1}^{PI*}$, $p_{2}^{NS*}>p_{2}^{PI*}$. (2) if $\tau_{1}<\tau_{2a}$, $q^{NS*}>q^{PI*}$.

where $\tau_{2a}=1-(\frac{a+\varepsilon }{a+\tau_{2}+\varepsilon }{)}^{3}$.

It shows that compared to the first subsidy, the second subsidy results in lower prices for core operators but higher prices for affiliated operators. In addition, when the subsidy rate of the first type of subsidy is below a certain threshold, the scale of consumer demand under the second type of subsidy is larger.

**Corollary 9:** (1) if $\tau_{1}<\tau_{2b}$, $u_{1}^{NS*}>u_{1}^{PI*}$, $k_{1}^{NS*}>k_{1}^{PI*}$; if $\tau_{1}>\tau_{2b}$, $u_{1}^{NS*}<u_{1}^{PI*}$, $k_{1}^{NS*}<k_{1}^{PI*}$. (2) $u_{2i}^{NS*}>u_{2i}^{PI*}$, $k_{2i}^{NS*}>k_{2i}^{PI*}$.

where $\tau_{2b}=1-\frac{{\left(a+\varepsilon \right)}^{2}}{{\left(a+\tau_{2}+\varepsilon \right)}^{2}}$.

It compares the investment situation under two types of subsidies. The results show that, firstly, as long as the subsidy rate meets $\tau_{1}<\tau_{2b}$, the second type of subsidy will increase the investment level of the core operator compared to the first type of subsidy. Secondly, regardless of the subsidy rate, the investment level of affiliated operators under the second type of subsidy is higher.

**Corollary 10:** (1) if $\tau_{1}<\tau_{2b}$, $\pi_{1}^{NS*}-\pi_{1}^{N*}=V_{1}^{NNS}>V_{1}^{NPI}=\pi_{1}^{PI*}-\pi_{1}^{N*}$.

(2) if $\tau_{1}<\tau_{2b}$ and $\tau_{1}<\tau_{1a}$, $\pi_{1}^{NS*}>\pi_{1}^{PI*}>\pi_{1}^{N*}$. if $\tau_{1}<\tau_{2b}$ and $\tau_{1}>\tau_{1a}$, $\pi_{1}^{NS*}>\pi_{1}^{N*}>\pi_{1}^{PI*}$

(3) if $n<n(\tau_{2})$, $\pi_{2i}^{NS*}-\pi_{2i}^{N*}=V_{2i}^{NNS}>V_{2i}^{NPI}=\pi_{2i}^{PI*}-\pi_{2i}^{N*}$, $\pi_{2i}^{NS*}>\pi_{2i}^{PI*}>\pi_{2i}^{N*}$.

if $n>n(\tau_{2})$, $\pi_{2i}^{NS*}-\pi_{2i}^{N*}=V_{2i}^{NNS}<V_{2i}^{NPI}=\pi_{2i}^{PI*}-\pi_{2i}^{N*}$, $\pi_{2i}^{PI*}>\pi_{2i}^{NS*}>\pi_{2i}^{N*}$.

where $n(\tau_{2})=(\frac{a+\tau_{2}+\varepsilon }{a+\varepsilon }{)}^{2}$.

(1) and (2) of the Corollary 10 reveal that when the subsidy rate satisfies the condition $\tau_{1}<\tau_{2b}$, compared to the first type of subsidy, the second type of subsidy leads to a more significant improvement in the profits of the core operator.

Among them, (3) indicates that for affiliated operators, as long as competition is not very incentivized, that is to say that the number of operators is below a certain threshold, the second subsidy leads to a more significant improvement in profits than the first subsidy. If the number of operators exceeds this threshold, there will be higher profit improvement under the first type of subsidy.

### 5.3 Numerical example

The above discussion has yielded many important conclusions. This subsection will visually present the results through data examples. The data for the main parameters comes from a survey of the STID project in Yaoshan Ancient Village, Libo County, Guizhou Province, China. This STID is based on traditional ethnic sports such as spinning and shooting to develop an STID. Currently, the annual reception volume is about 200000 people, and the expected maximum reception volume is about 500000 people. Therefore, the potential market demand is a=50. The capital of the two types of operators is expected to have a useful life of 10 years. Therefore, the depreciation rate $\delta_{1}=0.1$, $\delta_{2}=0.1$. After investigating the operating situation of the destination over the past few years, it was found that for every 10,000 yuan increase in fixed assets by the core operator and affiliated operators, the number of consumers increased by 300 and 100, respectively. Therefore, $\alpha_{1}=0.03$, $\alpha_{2}=0.01$. Currently, there are approximately 100 affiliated operators engaged in catering, accommodation, and other service work at the destination. Therefore, n=10. The current annual government subsidy that this destination can receive is approximately 300000 RMB. Therefore, T=3. In addition, the current risk-free interest rate is approximately 2%, therefore, ρ=0.02.

**Fig 2:**
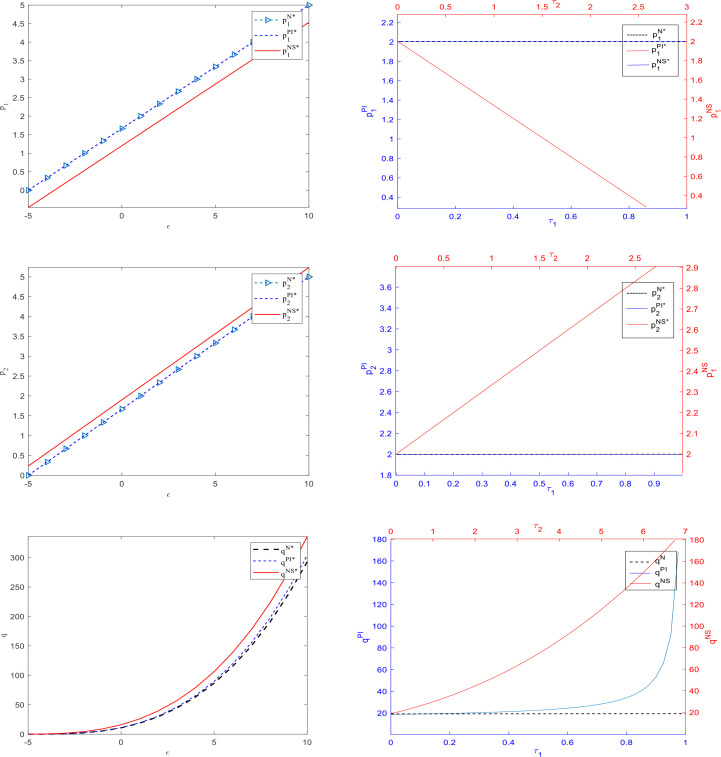
Changes of prices and number of consumers in subsidies rate and uncertain demand.

**Fig 3:**
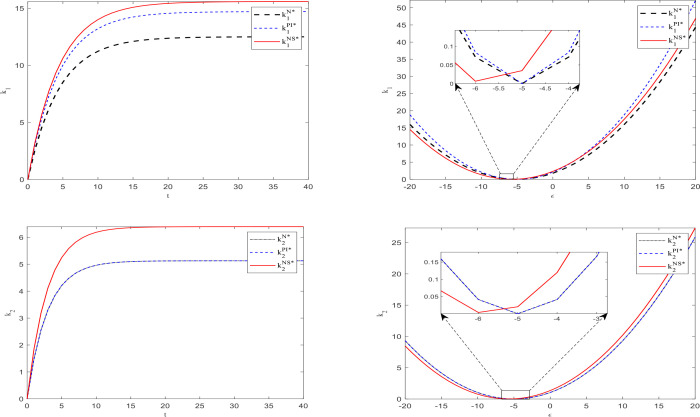
Chafignges in capital levels over time, demand disturbances, and subsidy rates.

**Fig 4:**
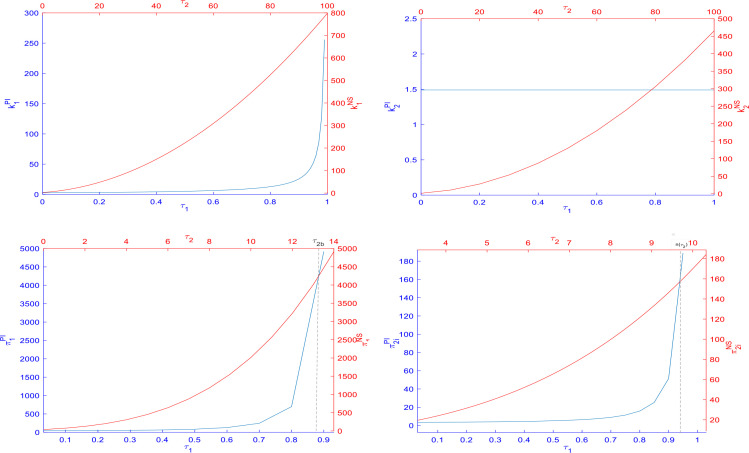
Impact of subsidies on the size of capital and on net income.

**Fig 5:**
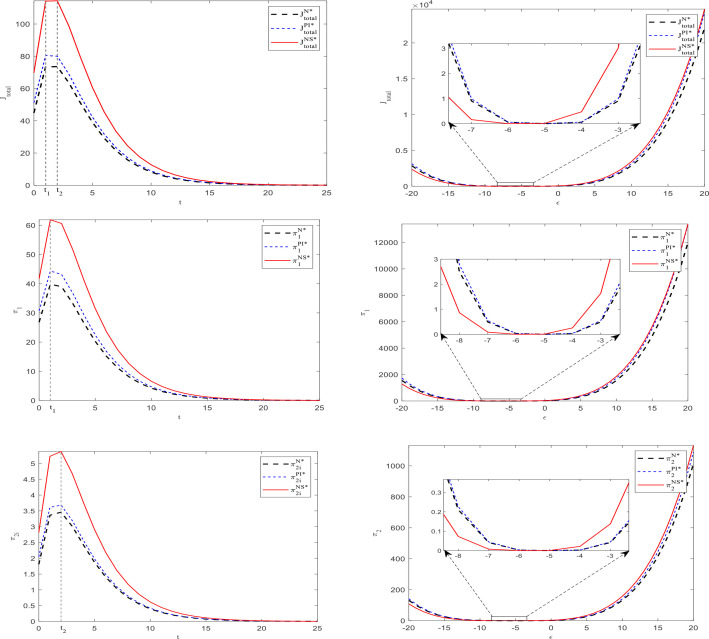
Variation of parties’ and total net benefits over time and demand perturbation parameters.

Based on the parameter assignments mentioned above and the equilibrium results obtained in the three scenarios, we used MATLAB software to obtain the data example shown in Figs 2–5.

Fig 2 shows that the prices of both core and affiliated operators are the same between two conditions: subsidizing the investment of the core operator and no subsidy. Under the subsidy based on the number of consumers, the prices of the core and affiliated operators show a downward and upward trend, respectively. The size of the consumer base is highest when subsidies are based on the number of consumers, followed by subsidies based on the core operator’s investment quantity, and lowest when there is no subsidy.

Additionally, random fluctuations in demand significantly impact prices and the number of consumers. Positive disturbances cause prices and quantities to rise synchronously, while negative disturbances have the opposite effect. These results are consistent with the relevant assertions in Corollaries 2, 4, 6, and 8.

Fig. 3 shows the changes in capital scale of two types of operators over time and demand disturbances in three scenarios. It indicates that for the core operator, its capital level is highest under the second type of subsidy (except when demand disturbances are very severe, which may result in the lowest level), followed by subsidies based on investment quantity, and lowest without subsidies. In terms of time dimension, the capital scale increases over time and eventually reaches a stable state (around the 25th period). It exhibits an inverted U-shape in response to demand disturbance, and excessive harmful demand disturbance may lead to a reversal of the capital level ranking in the three scenarios; that is, it may be the lowest under the second subsidy.

For affiliated operators, the capital size is equal in both cases, whether there is no subsidy or a subsidy based on the core operator investment. However, the capital level when subsidizing based on the number of consumers is higher than in the two aforementioned cases. The capital of these three situations increases over time and eventually reaches a stable state (around the 15th period). It exhibits an inverted U-shape in response to demand disturbance, and excessive harmful demand disturbance may lead to a reversal of the capital level ranking in the three scenarios, meaning it may be lower under the second subsidy.

The above results validate the relevant conclusions in Corollaries 5, 7, and 9.

Fig 4 shows the capital level and profit increment caused by two subsidy methods. In the figure, the horizontal and vertical axes of the blue coordinate system represent the subsidy rate and capital level, respectively, under the first subsidy method. Similarly, the two axes of the red coordinate system represent the corresponding indicators under the second subsidy method.

From the first row of data, it is evident that for core operators, their capital level increases as the subsidy rates of the two subsidy methods rise. Moreover, compared to the first subsidy method, the second subsidy method can enable core operators to obtain a higher level of capital. Additionally, under the first subsidy method, the capital level of affiliated operators remains unchanged, indicating that this subsidy method has no impact on the investment decisions of affiliated operators. However, under the second subsidy method, the capital of affiliated operators will increase in proportion to the increase in the subsidy rate. It reveals that the second subsidy method can better motivate both types of operators to increase investment, thereby expanding their capital scale and promoting the long-term stable development of STIDs. This result is entirely consistent with the viewpoint of Corollary 6.

In the second row of data, we also found that, except for the extreme case where the investment subsidy rate of core operators is close to or exceeds 0.9, the profit improvement level of the two types of operators is higher under the second subsidy method. The above findings validate the relevant conclusions in Corollary 10.

The first column of Fig 5 indicates that the net income and total net income of all parties will exhibit a trend of first increasing, reaching a peak, and then decreasing over time, ultimately reaching zero. This change pattern is consistent with the lifecycle of general construction projects, which includes stages such as rise, stable development, and decline.

Secondly, in terms of the impact of subsidy methods, the second method allows all parties to obtain higher net benefits compared to the first method. Without subsidies, the net income of all parties would be at the lowest level among the three scenarios.

The second column of Fig 5 shows the impact of demand disturbance on the net returns of all parties involved. When there is a significant harmful demand disturbance (e.g., demand disturbance parameter less than −5), it will change the standard ranking of net returns for all parties in the three scenarios, specifically manifested as the return rate under the second subsidy method reversing from the original first place to the third place. This situation is similar to the ranking changes in capital levels across the three scenarios depicted in Fig 4. The above findings strongly confirm the relevant statements of Corollary 7. Positive demand disturbances can correspondingly amplify the income-increasing effect of subsidies, while negative demand disturbances can weaken or even reverse the income-increasing effect of subsidy policies.

On the one hand, the parameter assignments used in this article are all derived from field investigations. On the other hand, the data examples and model calculation results mutually confirm each other. Therefore, the research conclusions of this article are robust.

## 6 Conclusions

### 6.1 Major findings

(1) When compared to a scenario with no subsidies, providing subsidies for the investment quantity of the core operator leads to an increase in both the core operator’s investment and the number of consumers in the STID. However, the prices set by both core and affiliate operators, as well as the investment quantity of affiliate operators, remain unchanged. Additionally, the profits of affiliate operators increase with a rise in the subsidy rate. In contrast, the profits of the core operator initially rise and then decline as the subsidy rate increases. If the subsidy rate exceeds a certain threshold, the core operator’s net profits decline rather than improve.
(2) In comparison to no subsidies, when subsidies are granted based on the performance (consumer scale) of the STID project, the investments and profits of both core and affiliate operators increase with the rising subsidy rate. Meanwhile, the prices set by the core operator decrease while those of affiliate operators increase.
(3) Random demand disturbances amplify the impact of subsidies on investment and profit growth. Specifically, under positive demand disturbances, the differences in investments and net incomes among participants across the three scenarios are magnified. Conversely, under negative demand disturbances, these differences in investments and profits among participants in the three scenarios are narrowed or even reversed.
(4) The number of affiliate operators influences the effect of subsidy policies on profit enhancement for affiliate operators. If the number of affiliate operators is below a certain threshold, the descending order of affiliate operators’ profits across the three scenarios is as follows: subsidy based on consumer quantity, subsidy based on public investment quantity, and no subsidy. If the number of affiliate operators exceeds this threshold, the order changes to subsidy based on investment quantity, subsidy based on consumer quantity, and no subsidy.

### 6.2 Managerial implications

(1) Both core and affiliate operators of the STID should determine their optimal strategies based on market demand conditions, government subsidy policies, and existing circumstances. For instance, if the government adopts a subsidy policy based on investment quantity, core operators should increase their investments to leverage government subsidies and lay a solid foundation for the project’s future development. Meanwhile, affiliate operators can capitalize on the increased consumer base resulting from the core operators’ higher investments to boost their revenues. If a subsidy policy based on consumer quantity is implemented, core operators should reduce service prices, increase investments, and expand the consumer base to secure as much subsidy as possible. In contrast, affiliate operators can seize this opportunity to raise prices, increase revenues, and also invest in enhancing service capabilities.
(2) For the government, the first step is to design the subsidy policy mechanism and select the most appropriate subsidy model. Generally, compared to subsidies based on investment quantity, subsidies based on operational performance result in a more extensive consumer base and higher profit levels for both types of operators. Therefore, it is advisable to choose the second subsidy model among the two.
(3) Assuming the subsidy model based on operational performance is selected, the subsidy rate should not be set too high, as an excessively high subsidy rate may not necessarily lead to increased profits for core operators.

### 6.3 Limitations and future research

Despite the high degree of novelty, realism, and managerial relevance of our study, it still has certain limitations. For example, we employed a linear demand function similar to previous studies, but the demand for STID may be non-linear. Additionally, although we considered the impact of uncertainty factors on consumer demand disturbances, we neglected other factors, such as the potential uncertainty in investment outcomes. We hope to address these shortcomings in future research.

## Supporting information

S1 Appendix (DOC)
