# Supplementary material for: Optimal subsidy design for sports tourism under demand uncertainty: A multi-agent strategic interaction framework
Source: PLoS One. 2025 Aug 29;20(8):e0329682. doi: 10.1371/journal.pone.0329682 (PMC12396729; doi:10.1371/journal.pone.0329682)
Supplement: S1 Appendix — (DOC) [file pone.0329682.s001.doc]

**Appendix**

Time t is omitted below for ease of writing

**Appendix A1**

**The proof for equilibrium results in the unsubsidized scenario (N)**

The following value function at time t can represent profit for each party in the STID: $\pi_{1}^{N*}=e^{-\rho t}V_{1}^{N}(k_{1}^{N},k_{2i}^{N})$ (A.1)

$\pi_{2i}^{N*}=e^{-\rho t}V_{2i}^{N}(k_{1}^{N},k_{2i}^{N})$ (A.2)

(A.3)

For any $k_{1}^{N}\geq0$and $k_{1}^{N}\geq0$,$V_{1}^{N}(k_{1}^{N},k_{2i}^{N})$and$V_{2i}^{N}(k_{1}^{N},k_{2i}^{N})$satisfy the Hamilton-Jacobi-Bellman function as

$\rho V_{1}^{N}(k_{1}^{N},k_{2i}^{N})=\max[p_{1}(a-p_{1}-p_{2}\text{+}\varepsilon)(\alpha_{1}k_{1}+\alpha_{2}nk_{2})-u_{1}^{2}(t)/2]+\frac{\partial V_{1}^{N}}{\partial k_{1}}(u_{1}-\delta_{1}k_{1})+\frac{\partial V_{1}^{N}}{\partial k_{2i}}(u_{2i}-\delta_{2}k_{2i})$ (A.4)

$\rho V_{_{2i}}^{N}(k_{1}^{N},k_{2i}^{N})=\max[p_{2}(a-p_{1}-p_{2}\text{+}\varepsilon)(\alpha_{1}k_{1}+\alpha_{2}nk_{2})/n-u_{2}^{2}(t)/2]+\frac{\partial V_{_{2i}}^{N}}{\partial k_{1}}(u_{1}-\delta_{1}k_{1})+\frac{\partial V_{_{2i}}^{N}}{\partial k_{2i}}(u_{2i}-\delta_{2}k_{2i})$ (A.5)

Thus, we obtain the following necessary conditions:

$\frac{\partial(\rho V_{1}^{N})}{\partial p_{1}}=0$, $\frac{\partial(\rho V_{1}^{N})}{\partial u_{1}}=0$, $\frac{\partial(\rho V_{_{2i}}^{N})}{\partial p_{2}}=0$, $\frac{\partial(\rho V_{_{2i}}^{N})}{\partial u_{2}}=0$

which respectively imply

$u_{1}=\frac{\partial V_{1}^{N}}{\partial k_{1}}$, $u_{2}=\frac{\partial V_{_{2i}}^{N}}{\partial k_{2}}$ (A.6)

$p_{1}=\frac{a-p_{2}\text{+}\varepsilon}{2}$, $p_{2}=\frac{a-p_{1}\text{+}\varepsilon}{2}$ (A.7)

By associating the two formulas for price in (A.7), we get

$p_{_{1}}^{N*}=\frac{a\text{+}\varepsilon}{3}$, $p_{_{2}}^{N*}=\frac{a\text{+}\varepsilon}{3}$ (A.8)

By substituting (A.6) and (A.8) into (A.4) and (A.5), we have

$\rho V_{_{1}}^{N}(k_{1}^{N},k_{2i}^{N})=[(\frac{a\text{+}\varepsilon}{3})^{2}\alpha_{1}-\delta_{1}\frac{\partial V_{_{1}}^{N}}{\partial k_{1}}]k_{1}+[(\frac{a\text{+}\varepsilon}{3})^{2}\alpha_{2}n-\delta_{2}\frac{\partial V_{_{1}}^{N}}{\partial k_{2i}}]k_{2i}+(\frac{\partial V_{_{1}}^{N}}{\partial k_{1}})^{2}/2+\frac{\partial V_{_{1}}^{N}}{\partial k_{2i}}\frac{\partial V_{_{2i}}^{N}}{\partial k_{2i}}$ (A.9) $\rho V_{_{2i}}^{N}(k_{1}^{N},k_{2i}^{N})=[(\frac{a\text{+}\varepsilon}{3})^{2}\frac{\alpha_{1}}{n}-\delta_{1}\frac{\partial V_{_{2i}}^{N}}{\partial k_{1}}]k_{1}+[(\frac{a\text{+}\varepsilon}{3})^{2}\alpha_{2}-\delta_{2}\frac{\partial V_{_{2i}}^{N}}{\partial k_{2i}}]k_{2i}+(\frac{\partial V_{_{2i}}^{N}}{\partial k_{2i}})^{2}/2+\frac{\partial V_{_{1}}^{N}}{\partial k_{1}}\frac{\partial V_{_{2i}}^{N}}{\partial k_{1}}$(A.10)

According to (A.9) and (A.10), we further infer that $V_{1}^{N}(k_{1}^{N},k_{2i}^{N})$ and $V_{2i}^{N}(k_{1}^{N},k_{2i}^{N})$are both linear about $k_{1}^{N}$and $k_{2i}^{N}$, thus, we set:

$\left\{\begin{matrix} V_{_{1}}^{N}=m_{1}^{N}k_{_{1}}^{N}+m_{2}^{N}k_{_{2i}}^{N}+m_{3}^{N} \\ V_{_{2i}}^{N}=r_{1}^{N}k_{_{1}}^{N}+r_{2}^{N}k_{_{2i}}^{N}+r_{3}^{N} \end{matrix}\right.$ (A.11)

where $m_{1}^{N}$, $m_{2}^{N}$, $r_{1}^{N}$, $r_{2}^{N}$ are constants. And, $\frac{\partial V_{_{1}}^{N}}{\partial k_{1}}=m_{1}^{N}$, $\frac{\partial V_{_{1}}^{N}}{\partial k_{2}}=m_{2}^{N}$, $\frac{\partial V_{_{2i}}^{N}}{\partial k_{1}}=r_{1}^{N}$, $\frac{\partial V_{_{2i}}^{N}}{\partial k_{2}}=r_{2}^{N}$. By substituting (A.11) into (A.9) and (A.10), we get $m_{1}^{N*}$, $m_{2}^{N*}$, $r_{1}^{N*}$, $r_{2}^{N*}$, $m_{3}^{N*}$, $r_{3}^{N*}$.

$\left\{\begin{matrix} m_{1}^{N*}=\frac{(a\text{+}\varepsilon)^{2}\alpha_{1}}{\text{9(1+}\delta_{1}\text{)}} \\ m_{2}^{N*}=\frac{(a\text{+}\varepsilon)^{2}\alpha_{2}n}{\text{9(1+}\delta_{2}\text{)}} \\ m_{3}^{N*}=(m_{1}^{N*})^{2}/2+m_{2}^{N*}r_{2}^{N*} \end{matrix}\right.$; $\left\{\begin{matrix} r_{1}^{N*}=\frac{(a\text{+}\varepsilon)^{2}\alpha_{1}}{9n\text{(1+}\delta_{1}\text{)}} \\ r_{2}^{N*}=\frac{(a\text{+}\varepsilon)^{2}\alpha_{2}}{\text{9(1+}\delta_{2}\text{)}} \\ r_{3}^{N\text{*}}=(r_{2}^{N*})^{2}/2+m_{1}^{N*}r_{1}^{N*} \end{matrix}\right.$ (A.12)

Substituting (A.12) and (A.13) into (A.6), we get the optimal equilibrium strategies:

$u_{_{1}}^{N*}=\frac{(a\text{+}\varepsilon)^{2}\alpha_{1}}{\text{9(1+}\delta_{1}\text{)}}$; $u_{_{2}}^{N*}=\frac{(a\text{+}\varepsilon)^{2}\alpha_{2}}{\text{9(1+}\delta_{2}\text{)}}$.

The calculation process in the second (PI) and third (NS) scenarios is similar to the above, so it is omitted here.

**The proof for Corollary 2**

The partial derivatives of price, demand, investment, and capital on demand disturbances for two types of operators in three scenarios yield the following results:

$\frac{\partial p_{_{1}}^{J*}}{\partial\varepsilon}=\frac{\partial p_{_{2}}^{J*}}{\partial\varepsilon}=\frac{1}{3}$, $\frac{\partial q^{J*}}{\partial\varepsilon}=\frac{1}{9}(\frac{(a\text{+}\varepsilon)^{2}\alpha_{1}^{2}}{\text{(1+}\delta_{1}\text{)}\delta_{1}}(1-e^{-\delta_{1}t})+\frac{(a\text{+}\varepsilon)^{2}n\alpha_{2}^{2}}{\text{(1+}\delta_{2}\text{)}\delta_{2}}(1-e^{-\delta_{2}t}))$, $\frac{\partial u_{_{1}}^{J*}}{\partial\varepsilon}=\frac{2(a\text{+}\varepsilon)\alpha_{1}}{\text{9(1+}\delta_{1}\text{)}}$,$\frac{\partial u_{2i}^{J*}}{\partial\varepsilon}=\frac{2(a\text{+}\varepsilon)\alpha_{2}}{\text{9(1+}\delta_{2}\text{)}}$, $\frac{\partial k_{_{1}}^{J*}}{\partial\varepsilon}=\frac{2(a\text{+}\varepsilon)\alpha_{1}(1-e^{-\rho t})}{\text{9(1+}\delta_{1}\text{)}\delta_{1}}$, $\frac{\partial k_{_{2i}}^{J*}}{\partial\varepsilon}=\frac{2(a\text{+}\varepsilon)\alpha_{2}(1-e^{-\rho t})}{\text{9(1+}\delta_{2}\text{)}\delta_{2}}$.

where $J=N,PI,NS$.

**The proof for Corollary 2**

(1) Calculate the partial derivative of the relevant variable $\tau_{1}$ to obtaint:

$\frac{\partial p_{_{1}}^{PI*}}{\partial\tau_{1}}=\frac{\partial p_{_{2}}^{PI*}}{\partial\tau_{1}}=\frac{\partial u_{_{2i}}^{PI*}}{\partial\tau_{1}}=\frac{\partial k_{_{2i}}^{PI*}}{\partial\tau_{1}}=0$, $\frac{\partial u_{_{1}}^{PI*}}{\partial\tau_{1}}=\frac{(a\text{+}\varepsilon)^{2}\alpha_{1}}{9(1-\tau_{1})^{2}\text{(1+}\delta_{1}\text{)}}$, $\frac{\partial k_{_{1}}^{PI*}}{\partial\tau_{1}}=\frac{(a\text{+}\varepsilon)^{2}\alpha_{1}}{9(1-\tau_{1})^{2}\text{(1+}\delta_{1}\text{)}\delta_{1}}(1-e^{-\delta_{1}t})$, $\frac{\partial q^{PI*}}{\partial\tau_{1}}=\frac{(a\text{+}\varepsilon)^{3}\alpha_{1}^{2}}{27(1-\tau_{1})^{2}\text{(1+}\delta_{1}\text{)}\delta_{1}}(1-e^{-\delta_{1}t})$, $\frac{\partial\pi_{2i}^{PI*}}{\partial\tau_{1}}=\frac{(1-\delta_{1})(a\text{+}\varepsilon)^{4}\alpha_{1}^{2}e^{-\rho t}}{\delta_{1}81n(1-\tau_{1})^{2}\text{(1+}\delta_{1}\text{)}^{2}}$.

$a$, $\varepsilon$, $\alpha_{1}$, $\delta_{1}$, $\alpha_{2}$, $\delta_{2}$, $n$, $1-e^{-\delta_{1}t}$, $1-e^{-\delta_{2}t}$, $1-\delta_{1}$, $1-\tau_{1}$are greater than zero. Therefore, $\frac{\partial u_{_{1}}^{PI*}}{\partial\tau_{1}}>0$, $\frac{\partial k_{_{1}}^{PI*}}{\partial\tau_{1}}>0$. $\frac{\partial q^{PI*}}{\partial\tau_{1}}>0$, $\frac{\partial\pi_{2i}^{PI*}}{\partial\tau_{1}}>0$.

(2) Beacause: $\frac{\partial\pi_{1}^{PI*}}{\partial\tau_{1}}=[\frac{(1-\tau_{1})(1+\delta_{1})-1}{(1-\tau_{1})^{2}\delta_{1}}](m_{1}^{PI*})^{2}e^{-\rho t}$.

If $(1-\tau_{1})(1+\delta_{1})-1>0$, i.e.,$0<\tau_{1}<\frac{\delta_{1}}{1+\delta_{1}}$, we get $\frac{\partial\pi_{1}^{PI*}}{\partial\tau_{1}}>0$; if $\frac{\delta_{1}}{1+\delta_{1}}<\tau_{1}<1$,we have $\frac{\partial\pi_{1}^{PI*}}{\partial\tau_{1}}<0$.

**The proof for Corollary 3**

Because:$\frac{\partial p_{_{1}}^{PI*}}{\partial\varepsilon}=\frac{\partial p_{_{2i}}^{PI*}}{\partial\varepsilon}=\frac{1}{3}$, $\frac{\partial q^{PI*}}{\partial\varepsilon}=\frac{2(a\text{+}\varepsilon)\alpha_{1}}{9(1-\tau_{1})\text{(1+}\delta_{1}\text{)}\delta_{1}}(1-e^{-\delta_{1}t})+\frac{2(a\text{+}\varepsilon)\alpha_{2}}{\text{9(1+}\delta_{2}\text{)}\delta_{2}}(1-e^{-\delta_{2}t})$, $\frac{\partial u_{_{1}}^{PI*}}{\partial\varepsilon}=\frac{2(a\text{+}\varepsilon)\alpha_{1}}{9(1-\tau_{1})\text{(1+}\delta_{1}\text{)}}$, $\frac{\partial u_{_{2i}}^{PI*}}{\partial\varepsilon}=\frac{2(a\text{+}\varepsilon)\alpha_{2}}{\text{9(1+}\delta_{2}\text{)}}$, $\frac{\partial k_{_{1}}^{PI*}}{\partial\varepsilon}=\frac{2(a\text{+}\varepsilon)\alpha_{1}}{9(1-\tau_{1})\text{(1+}\delta_{1}\text{)}\delta_{1}}(1-e^{-\delta_{1}t})$, $\frac{\partial k_{_{2i}}^{PI*}}{\partial\varepsilon}=\frac{2(a\text{+}\varepsilon)\alpha_{2}(1-e^{-\rho t})}{\text{9(1+}\delta_{2}\text{)}\delta_{2}}$.

$a$, $\varepsilon$, $\alpha_{1}$, $\delta_{1}$, $\alpha_{2}$, $\delta_{2}$, $n$, $1-e^{-\delta_{1}t}$, $1-e^{-\delta_{2}t}$, $1-\delta_{1}$, $1-\tau_{1}$are greater than zero to ensure that the above deviation results are positive.

**Appendix C3**

**The proof for Corollary 4**

$\frac{\partial p_{_{1}}^{NS*}}{\partial\tau_{2}}=-\frac{2}{3}$, $\frac{\partial p_{_{2}}^{NS*}}{\partial\tau_{2}}=\frac{1}{3}$, $\frac{\partial q^{NS*}}{\partial\tau_{2}}=\frac{1}{3}(\frac{(a+\tau_{2}\text{+}\varepsilon)^{2}\alpha_{1}^{2}}{\text{(1+}\delta_{1}\text{)}\delta_{1}}(1-e^{-\delta_{1}t})+\frac{(a+\tau_{2}\text{+}\varepsilon)^{2}n\alpha_{2}^{2}}{\text{9(1+}\delta_{2}\text{)}\delta_{2}}(1-e^{-\delta_{2}t}))$,

$\frac{\partial u_{_{1}}^{NS*}}{\partial\tau_{2}}=\frac{2(a+\tau_{2}\text{+}\varepsilon)\alpha_{1}}{\text{9(1+}\delta_{1}\text{)}}$,$\frac{\partial u_{_{2i}}^{NS*}}{\partial\tau_{2}}=\frac{2(a+\tau_{2}\text{+}\varepsilon)\alpha_{2}}{\text{9(1+}\delta_{2}\text{)}}$,$\frac{\partial k_{_{1}}^{NS*}}{\partial\tau_{2}}=\frac{2(a+\tau_{2}\text{+}\varepsilon)\alpha_{1}}{\text{9(1+}\delta_{1}\text{)}\delta_{1}}(1-e^{-\delta_{1}t})$,$\frac{\partial k_{_{2i}}^{NS*}}{\partial\tau_{2}}=\frac{2(a+\tau_{2}\text{+}\varepsilon)\alpha_{2}}{\text{9(1+}\delta_{2}\text{)}\delta_{2}}(1-e^{-\delta_{2}t})$, $\frac{\partial\pi_{1}^{NS*}}{\partial\tau_{2}}=\frac{4(a+\tau_{2}\text{+}\varepsilon)^{3}\alpha_{1}^{2}}{\text{81(1+}\delta_{1}\text{)}^{2}\delta_{1}}(1-e^{-\delta_{1}t}+4.5\delta_{1})+\frac{4(a+\tau_{2}\text{+}\varepsilon)^{3}\alpha_{2}^{2}n}{\text{81(1+}\delta_{2}\text{)}^{2}\delta_{2}}(1-e^{-\delta_{2}t}+\delta_{2})$,

$\frac{\partial\pi_{2i}^{NS*}}{\partial\tau_{2}}=\frac{4(a+\tau_{2}\text{+}\varepsilon)^{3}\alpha_{1}^{2}}{81n\text{(1+}\delta_{1}\text{)}^{2}\delta_{1}}(1-e^{-\delta_{1}t}+\delta_{1})+\frac{4(a+\tau_{2}\text{+}\varepsilon)^{3}\alpha_{2}^{2}}{\text{81(1+}\delta_{2}\text{)}^{2}\delta_{2}}(1-e^{-\delta_{2}t}+4.5\delta_{2})$.

**The proof for Corollary 5**

We get $p_{1}^{PI*}=p_{_{1}}^{N*}$, $p_{2}^{PI*}=p_{_{2}}^{N*}$, $u_{_{2i}}^{PI*}=u_{_{2i}}^{N*}$, $k_{_{2i}}^{PI*}=k_{_{2i}}^{N*}$,

$u_{_{1}}^{PI*}-u_{_{1}}^{N*}=\frac{\tau_{1}(a\text{+}\varepsilon)^{2}\alpha_{1}}{9(1-\tau_{1})\text{(1+}\delta_{1}\text{)}}$, $k_{_{1}}^{PI*}-k_{_{1}}^{N*}=\frac{\tau_{1}(a\text{+}\varepsilon)^{2}\alpha_{1}}{9(1-\tau_{1})\text{(1+}\delta_{1}\text{)}\delta_{1}}(1-e^{-\delta_{1}t})$.

Similarly, the corresponding parameter variables in the above equation are all positive. Therefore, $u_{_{1}}^{PI*}>u_{_{1}}^{N*}$and $k_{_{1}}^{PI*}>k_{_{1}}^{N*}$.

**The proof for Corollary 6**

(1) The difference between $\pi_{_{1}}^{PI*}$and $\pi_{_{1}}^{N*}$is:

$V_{1}^{NPI}\text{=}\pi_{_{1}}^{PI*}-\pi_{_{1}}^{N*}=[\frac{2(1-e^{-\delta_{1}t})-\tau_{1}(2(1-e^{-\delta_{1}t})+\delta_{1})}{2(1-\tau_{1})\delta_{1}}](\frac{(a\text{+}\varepsilon)^{2}\alpha_{1}}{\text{9(1+}\delta_{1}\text{)}})^{2}\frac{\tau_{1}}{(1-\tau_{1})}$.Obviously, $V_{1}^{NPI}\text{>0}$depends on$2(1-e^{-\delta_{1}t})-\tau_{1}(2(1-e^{-\delta_{1}t})+\delta_{1})$: when$2(1-e^{-\delta_{1}t})-\tau_{1}(2(1-e^{-\delta_{1}t})+\delta_{1})>0$, i.e., $\tau_{1}<\tau_{1a}$,we have$\pi_{_{1}}^{PI*}>\pi_{_{1}}^{N*}$; when $2(1-e^{-\delta_{1}t})-\tau_{1}(2(1-e^{-\delta_{1}t})+\delta_{1})<0$, i.e., $\tau_{1}>\tau_{1a}$,we get$\pi_{_{1}}^{PI*}<\pi_{_{1}}^{N*}$.

(2) The difference between $\pi_{_{2i}}^{PI*}$and $\pi_{_{2i}}^{N*}$is: $V_{2i}^{NPI}=\pi_{_{2i}}^{PI*}-\pi_{_{2i}}^{N*}=\frac{\tau_{1}(1-e^{-\delta_{1}t}+\delta_{1})}{n(1-\tau_{1})\delta_{1}})(\frac{(a\text{+}\varepsilon)^{2}\alpha_{1}}{\text{9(1+}\delta_{1}\text{)}})^{2}$. The corresponding parameter variables in the above equation are all positive. Therefore, $\pi_{_{2i}}^{PI*}>\pi_{_{2i}}^{N*}$.

(3) The difference between $J_{_{total}}^{PI*}$and $J_{_{total}}^{N*}$is:

$$V_{^{total}}^{NPI}\text{=}J_{_{total}}^{PI*}-J_{_{total}}^{N*}=[\frac{4(1-\tau_{1})(1-e^{-\delta_{1}t})+2\delta_{1}-3\tau_{1}\delta_{1}}{2(1-\tau_{1})\delta_{1}}](\frac{(a\text{+}\varepsilon)^{2}\alpha_{1}}{\text{9(1+}\delta_{1}\text{)}})^{2}\frac{\tau_{1}}{(1-\tau_{1})}$$

If $4(1-\tau_{1})(1-e^{-\delta_{1}t})+2\delta_{1}-3\tau_{1}\delta_{1}>0$, i.e., $\tau_{1}<\tau_{1b}$, we get $V_{^{total}}^{NPI}=V_{^{total}}^{PI}-V_{^{total}}^{N}>0$; if

$4(1-\tau_{1})(1-e^{-\delta_{1}t})+2\delta_{1}-3\tau_{1}\delta_{1}<0$, i.e., $\tau_{1}>\tau_{1b}$, we get $V_{^{total}}^{NPI}=V_{^{total}}^{PI}-V_{^{total}}^{N}<0$

(4) The effect of the subsidy rate on net income of the FST destination is:

$\frac{\partial V_{^{total}}^{NPI}}{\partial\tau_{1}}=\frac{1}{2(1-\tau_{1})^{2}}\frac{(a\text{+}\varepsilon)^{2}\alpha_{1}}{\text{9(1+}\delta_{1}\text{)}})^{2}[\frac{4(1-e^{-\delta_{1}t})}{\delta_{1}}+\frac{2-4\tau_{1}}{(1-\tau_{1})}]$. Similarly, if $\tau_{1}<\frac{2(1-e^{-\delta_{1}t})+\delta_{1}}{2(1-e^{-\delta_{1}t}+\delta_{1})}$, we obtain $\frac{\partial V_{^{total}}^{NPI}}{\partial\tau_{1}}>0$; if $\tau_{1}>\frac{2(1-e^{-\delta_{1}t})+\delta_{1}}{2(1-e^{-\delta_{1}t}+\delta_{1})}$, we obtain $\frac{\partial V_{^{total}}^{NPI}}{\partial\tau_{1}}<0$.

**The proof for Corollary 7**

We have: $p_{_{1}}^{NS*}-p_{_{1}}^{N*}=\frac{-2\tau_{2}}{3}$, $p_{_{2}}^{NS*}-p_{_{2}}^{N*}=\frac{\tau_{2}}{3}$, $q^{NS*}-q^{N*}=\frac{(a\text{+}\tau_{2}\text{+}\varepsilon)(\alpha_{1}k_{^{1}}^{^{NS*}}+\alpha_{2}nk_{^{2}}^{^{NS*}})-(a\text{+}\varepsilon)(\alpha_{1}k_{^{1}}^{^{N*}}+\alpha_{2}nk_{^{2}}^{^{N*}})}{3}$, $u_{_{1}}^{NS*}-u_{_{1}}^{N*}=\frac{(2a+\tau_{2}\text{+2}\varepsilon)(a\text{+}\varepsilon)\alpha_{1}}{\text{9(1+}\delta_{1}\text{)}}$,$u_{_{2i}}^{NS*}-u_{_{2i}}^{N*}=\frac{(2a+\tau_{2}\text{+2}\varepsilon)(a\text{+}\varepsilon)\alpha_{2}}{\text{9(1+}\delta_{2}\text{)}}$,

$k_{_{1}}^{NS*}-k_{_{1}}^{N*}=\frac{(2a+\tau_{2}\text{+2}\varepsilon)(a\text{+}\varepsilon)\alpha_{1}(1-e^{-\delta_{1}t})}{\text{9(1+}\delta_{1}\text{)}\delta_{1}}$, $k_{_{2i}}^{NS*}-k_{_{2i}}^{N*}=\frac{(2a+\tau_{2}\text{+2}\varepsilon)(a\text{+}\varepsilon)\alpha_{1}(1-e^{-\delta_{2}t})}{\text{9(1+}\delta_{2}\text{)}\delta_{2}}$. It is obvious that the corresponding parameter variables in the above equation are all positive except $p_{_{1}}^{NS*}-p_{_{1}}^{N*}$.

**The proof for Corollary 8**

(1) The differences between $p_{_{1}}^{NS*}$ and $p_{1}^{PI*}$, $p_{_{2}}^{NS*}$ and $p_{2}^{PI*}$are respectively:

$p_{_{1}}^{NS*}-p_{_{1}}^{PI*}=\frac{-2\tau_{2}}{3}$, $p_{_{2}}^{NS*}-p_{_{2}}^{PI*}=\frac{\tau_{2}}{3}$.Therefore, we have $p_{_{1}}^{NS*}<p_{_{1}}^{PI*}$, $p_{_{2}}^{NS*}>p_{_{2}}^{PI*}$for $\tau_{2}>0$.

(2) The difference between $q^{NS*}$and $q^{PI*}$is:

$q^{NS*}-q^{PI*}=\frac{(a\text{+}\varepsilon)[\alpha_{1}(k_{1}^{NS*}-k_{1}^{PI*})+\alpha_{2}n(k_{_{2i}}^{NS*}-k_{_{2i}}^{PI*})]\text{+}\tau_{2}(\alpha_{1}k_{1}^{NS*}+\alpha_{2}nk_{_{2i}}^{NS*})}{3}$. We get: if $\tau_{1}<\tau_{2a}$, $q^{NS*}>q^{PI*}$.

**The proof for Corollary 9**

(1) The differences between $u_{_{1}}^{NS*}$and $u_{1}^{PI*}$, $k_{_{1}}^{NS*}$and $k_{1}^{PI*}$are respectively:

$u_{_{1}}^{NS*}-u_{_{1}}^{PI*}=(1-\tau_{1})(2(a\text{+}\varepsilon)\tau_{2}+\tau_{2}^{2})-\tau_{1}(a\text{+}\varepsilon)^{2}$, $k_{_{1}}^{NS*}-k_{_{1}}^{PI*}=[(1-\tau_{1})(2(a\text{+}\varepsilon)\tau_{2}+\tau_{2}^{2})-\tau_{1}(a\text{+}\varepsilon)^{2}](1-e^{-\delta_{1}t})$.

where$(1-\tau_{1})(2(a\text{+}\varepsilon)\tau_{2}+\tau_{2}^{2})-\tau_{1}(a\text{+}\varepsilon)^{2}>0$, i.e., $\tau_{1}<\frac{2(a\text{+}\varepsilon)\tau_{2}+\tau_{2}^{2}}{(a\text{+}\varepsilon+\tau_{2})^{2}}$, we get$u_{_{1}}^{NS*}>u_{_{1}}^{PI*}$,$k_{_{1}}^{NS*}>k_{_{1}}^{PI*}$; where $(1-\tau_{1})(2(a\text{+}\varepsilon)\tau_{2}+\tau_{2}^{2})-\tau_{1}(a\text{+}\varepsilon)^{2}<0$,i.e., $\tau_{1}>\frac{2(a\text{+}\varepsilon)\tau_{2}+\tau_{2}^{2}}{(a\text{+}\varepsilon+\tau_{2})^{2}}$, we get$u_{_{1}}^{NS*}<u_{_{1}}^{PI*}$, $k_{_{1}}^{NS*}<k_{_{1}}^{PI*}$.

(2) The differences between $u_{_{2i}}^{NS*}$and $u_{_{2i}}^{PI*}$, $k_{_{2i}}^{NS*}$and $k_{_{2i}}^{N*}$are respectively:

$u_{_{2i}}^{NS*}-u_{_{2i}}^{PI*}=\frac{(2a+\tau_{2}\text{+2}\varepsilon)(a\text{+}\varepsilon)\alpha_{2}}{\text{9(1+}\delta_{2}\text{)}}$, $k_{_{2i}}^{NS*}-k_{_{2i}}^{PI*}=\frac{(2a+\tau_{2}\text{+2}\varepsilon)(a\text{+}\varepsilon)\alpha_{2}(1-e^{-\delta_{2}t})}{\text{9(1+}\delta_{2}\text{)}}$.

The corresponding parameter variables in the above equation are all positive. Therefore, $u_{_{2i}}^{NS*}>u_{_{2i}}^{PI*}$and $k_{_{2i}}^{NS*}>k_{_{2i}}^{PI*}$.

**The proof for Corollary 10**

(1) The differences between $V_{_{1}}^{NNS}$and $V_{1}^{NPI}$is:

$$\begin{matrix} V_{_{1}}^{NNS}-V_{_{1}}^{NPI}=\frac{((a+\tau_{2}\text{+}\varepsilon)^{4}(1-\tau_{1})^{2}-(a\text{+}\varepsilon)^{4})\alpha_{1}^{2}(1-e^{-\delta_{1}t})}{81(1-\tau_{1})^{2}\text{(1+}\delta_{1}\text{)}^{2}\delta_{1}}+\frac{((a+\tau_{2}\text{+}\varepsilon)^{4}-(a\text{+}\varepsilon)^{4})n\alpha_{2}^{2}(1-e^{-\delta_{2}t})}{\text{81(1+}\delta_{2}\text{)}^{2}\delta_{2}} \\ +G(\tau_{1})(\frac{\alpha_{1}}{\text{9(1+}\delta_{1}\text{)}})^{2}+\frac{((a+\tau_{2}\text{+}\varepsilon)^{4}-(a\text{+}\varepsilon)^{4})n\alpha_{2}^{2}}{\text{81(1+}\delta_{2}\text{)}^{2}} \end{matrix}$$

Where $G(\tau_{1})=(\frac{(a+\tau_{2}\text{+}\varepsilon)^{4}}{2}-\frac{1}{(1-\tau_{1})^{2}}+\frac{1}{2(1-\tau_{1})^{4}})$. Therefore, $V_{1}^{NNS}-V_{1}^{NPI}>0$depends on $G(\tau_{1})>0$, i.e., $\tau_{1}<1-\frac{(a\text{+}\varepsilon)^{2}}{(a+\tau_{2}\text{+}\varepsilon)^{2}}$.

(2) The differences between $V_{2i}^{NNS}$and $V_{2i}^{NPI}$is:

$$\begin{matrix} V_{2i}^{NNS}-V_{2i}^{NPI}=\frac{((a+\tau_{2}\text{+}\varepsilon)^{4}-n(a\text{+}\varepsilon)^{4})\alpha_{1}^{2}(1-e^{-\delta_{1}t})}{81n(1-\tau_{1})^{2}\text{(1+}\delta_{1}\text{)}^{2}\delta_{1}}+\frac{((a+\tau_{2}\text{+}\varepsilon)^{4}-n^{2}(a\text{+}\varepsilon)^{4})\alpha_{2}^{2}(1-e^{-\delta_{2}t})}{\text{81(1+}\delta_{2}\text{)}^{2}\delta_{2}} \\ +\frac{((a+\tau_{2}\text{+}\varepsilon)^{4}-(a\text{+}\varepsilon)^{4})\alpha_{2}^{2}}{\text{126(1+}\delta_{2}\text{)}^{2}}+G(\tau_{2}) \end{matrix}$$

Where $G(\tau_{2})=\frac{(a+\tau_{2}\text{+}\varepsilon)^{4}\alpha_{1}^{2}}{81n\text{(1+}\delta_{1}\text{)}^{2}}-\frac{(a\text{+}\varepsilon)^{4}\alpha_{1}^{2}}{81n(1-\tau_{1})^{2}\text{(1+}\delta_{1}\text{)}^{2}}$. By means of the deflation method, we get Corollary 10 (3).
